# Successful CyberKnife Irradiation of 1000 cc Hemicranial Meningioma: 6-year Follow-up

**DOI:** 10.7759/cureus.384

**Published:** 2015-11-20

**Authors:** Mikhail Galkin, Andrey V. Golanov, Natalia Antipina, Gennady Gorlachev

**Affiliations:** 1 Department of Radiation Oncology, Burdenko Neurosurgical Institute

**Keywords:** meningioma, hypo-fractionated radiotherapy, cyberknife©, radiosurgery

## Abstract

Meningiomas are common benign tumors with accepted treatment approaches and usually don't challenge healthcare specialists. We present a case of a huge unresectable hemicranial meningioma, which was successfully treated with hypofractionated irradiation.

A male patient, sixty-two years of age, suffered for over 12 years from headaches, facial deformity, right eye displacement, right eye movement restriction, right-sided ptosis, and facial hypoesthesia. MRI and CT studies revealed an extended hemicranial meningioma. Prior to irradiation, the patient underwent four operations. Eventually, the tumor was irradiated with the CyberKnife in August 2009. Tumor volume composed 1085 cc. The mean dose of 35.3 Gy was delivered in 7 fractions (31.5 Gy at 72% isodose line comprising 95% of tumor volume). The patient was followed during six years and experienced only mild (Grade 1-2 CTCAE) acute skin and mucosa reactions. During the follow-up period, we observed target volume shrinkage for 17% (for 26% after excluding hyperostosis) and regression of intracranial hypertension signs.

Due to the extreme volume and complex shape of the tumor, spreading along the surface of the hemisphere as well as an optic nerve involvement, the case presented would not be generally considered suitable for irradiation, especially for hypofractionation. We regard this clinical situation not as a treatment recommendation, but as a demonstration of the underestimated possibilities of hypofractionation regimen and CyberKnife system, both of which are limited with our habit of conventional treatments.

## Introduction

Meningiomas are common benign tumors, which usually can be successfully managed with standard treatment options (surgery or/and irradiation). Contemporary surgical and irradiation methods provide high tumor growth control rates with low morbidity and mortality [1-3].

Nevertheless, despite extensive clinical experience, there are situations when standard approaches are inapplicable. In such cases, healthcare specialists rely on their previous experience, on their assessment of the clinical situation, and some radiobiological calculations to provide optimal treatment.

In this paper, we present a case of successful CyberKnife hypofractionated irradiation of a large complex-shaped meningioma.

## Case presentation

In 2009, a 62-year-old male patient was referred to the Radiation Oncology Department of the Burdenko Neurosurgical Institute. He had been previously treated with consecutive surgical resections of a right-sided cranio-orbital meningioma in 1998, 2000, 2001, and 2009. All histological investigations showed a typical WHO Grade I meningioma with an infiltrative growth pattern. At the time of admission, he had headaches, facial deformity, right eye displacement, right eye movement restriction, right-sided ptosis, and right-sided facial hypoesthesia. Signs of intracranial hypertension were identified with an eye fundus study. Informed patient consent was obtained for treatment.

MRI with contrast enhancement revealed right-sided extended calvarial, skull base, and facial meningioma. The tumor caused calvarial, skull base, and facial hyperostosis, infiltrated chewing muscles, and thickened meninges and periosteum, up to 15 mm. Due to massive unilateral meninges thickening, septa pellucida displacement was found.

**Figure 1:**
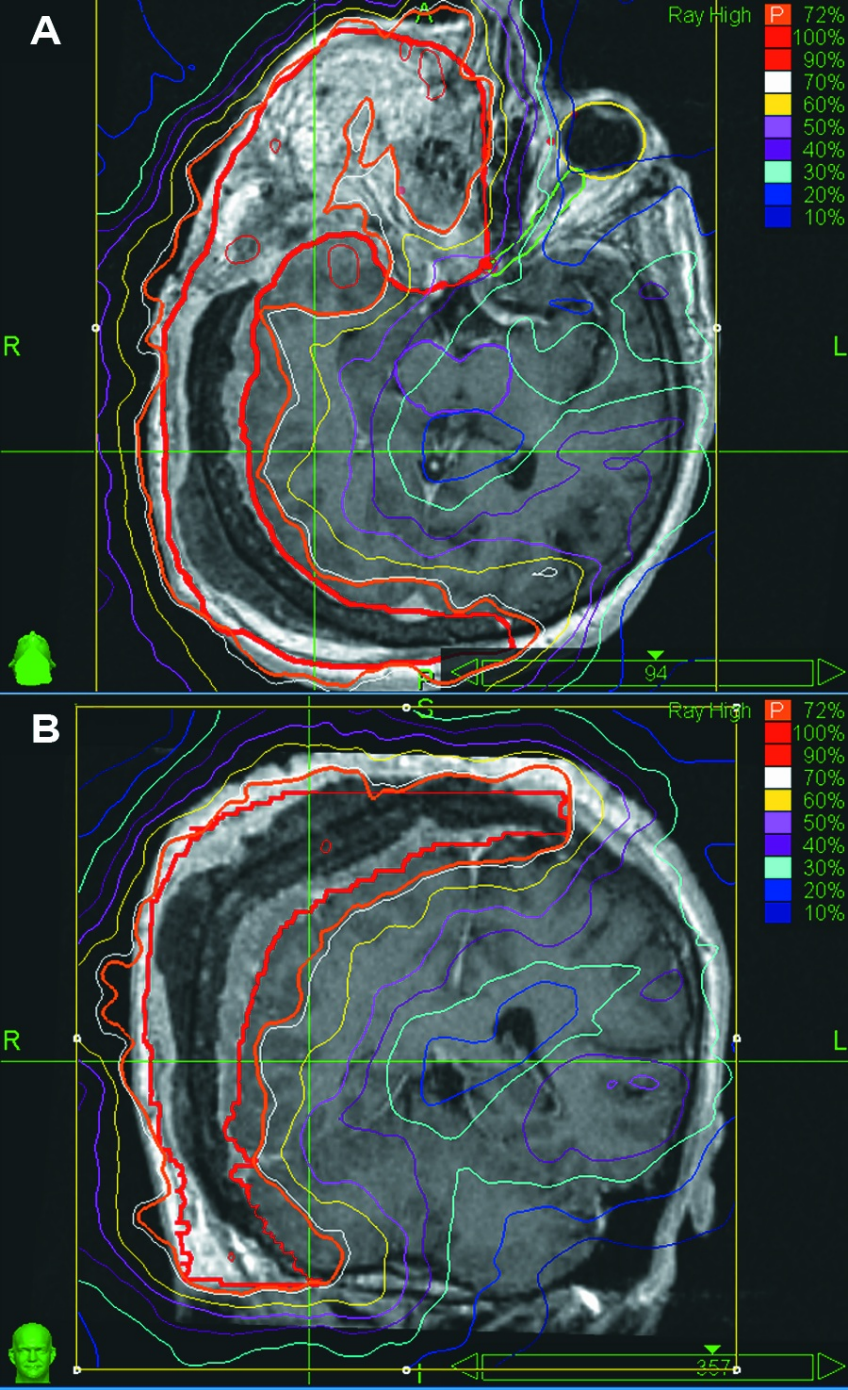
Tumor extent with MRI and isodose distributions A - axial plane, B - coronal plane

The patient was treated with the CyberKnife. Tumor volume composed 1085 cc. Due to hyperostotic changes of the skull, the target included both soft tissue (702 cc) and bone components (383 cc). The following treatment regimen was chosen: 35 Gy total median dose in 7 fractions with 48-72 hours interfraction interval. Two conic collimators with 50 and 25 mm aperture diameter were chosen for treatment delivery. The acceptable treatment plan was generated after several cycles of alternation of iterative and simplex algorithms (Table 1). It contained 271 beams. The mean dose was 35.3 Gy. The dose of 31.5 Gy was achieved at the 72% isodose line, encompassing 95% of the target volume (Figure 1). Irradiation was performed from August 19 to September 2, 2009. During treatment time, and several days after, the patient was administered dexamethasone (up to 16 mg daily) and acetazolamidum.

**Table 1:** Treatment parameters

Parameter	Value
Target volume, cc	1085
Dose for 95% of target volume, Gy	31.5
Mean target dose, Gy	35.3
Maximum target dose, Gy	43.8
Minimum target dose, Gy	0.4
Isodose covering 95% of target volume, %	72
New Conformity Index	1.61
Homogeneity Index	1.39
Coverage, %	95.62

During the month after treatment, mild acute adverse reactions were detected. The following reactions were noticed: alopecia (Grade 2 CTCAE 4.02), skin hyperemia (Grade 1, CTCAE 4.02), and oral mucositis (Grade 2 CTCAE 4.02). All reactions regressed spontaneously or with a short course of medications.

The patient was followed for six years after his treatment with consecutive MRI studies and clinical evaluations. No late reactions were detected during this period. After treatment, the patient noticed headaches ceasing and partial regression of his facial deformation. Follow-up neuro-ophthalmological evaluation six months after treatment detected regression of the intracranial hypertension signs.

**Figure 2:**
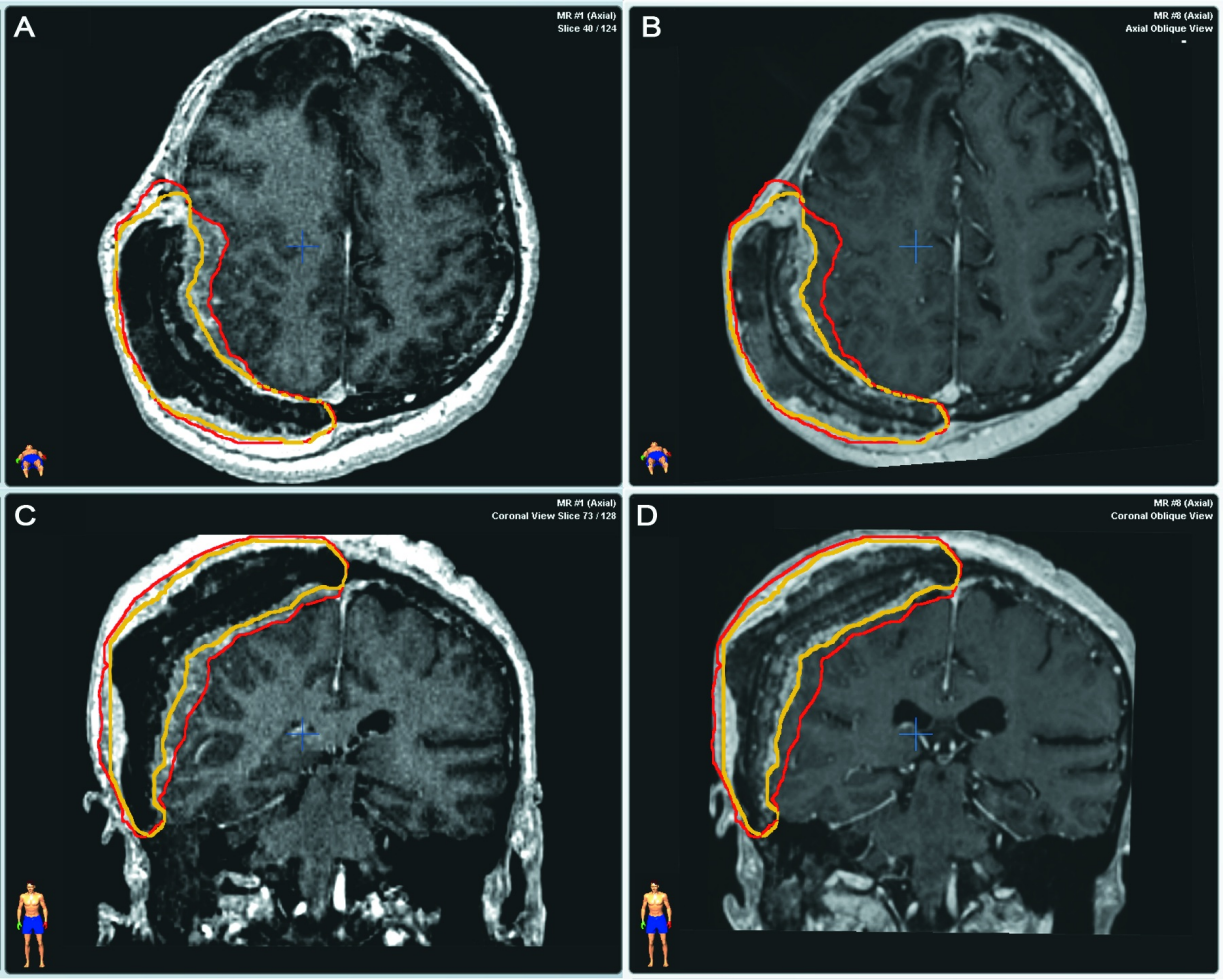
Tumor extent with MRI before and 6 years after irradiation Before irradiation - A (axial plane), C (coronal plane)/ 6 years after treatment - B (axial plane), D (coronal plane). Color lines delineate tumor contours before (red line) and after treatment (yellow line).

Follow-up images revealed tumor shrinkage six years after the treatment (Figure 2). Tumor shrinkage was obvious with image fusion performed with iPlan software (BrainLab, Germany). Target volume diminished from 1085 cc to 901 cc (for 17%). Simultaneously, the soft-tissue component decreased from 702 cc to 518 cc (for 26%).  

## Discussion

We presented a rare case of a huge meningioma invading half of the skull. Despite extreme volume and extent, the tumor caused only mild symptoms and slightly affected life quality. We supposed this was due to the slow growth rate. Tumor extent, benign behavior, and patient status were regarded as contraindications for surgery. After interdisciplinary consultation, we proposed irradiation. To our knowledge, it is the only published case of hypofractionated irradiation of such a large intra-extracranial tumor.

Because of the uniqueness of the clinical situation, the most difficult part was to select an optimal treatment facility and treatment regimen.

Due to department equipment with Gamma Knife, CyberKnife, Novalis with ExacTrac, and Primus, we had different opportunities for treatment delivery. CyberKnife was chosen due to several reasons. The first was irradiation with multiple non-isocentric, non-coplanar beams. The beams could be directed tangentially for more favorable dose distribution in this roughly hemispherical tumor. The second reason was the inverse treatment planning algorithm, which was favorable in this complex tumor. Hypofractionated irradiation with the CyberKnife was demonstrated to be effective for meningiomas [4-5]. We supposed that IMRT with common LinAcs with micromultileaf collimators could also be regarded in this situation.

Our department practice is to prescribe the mean dose. The mean dose of 35 Gy in 7 fractions was assigned. After treatment planning, it was 31 Gy for 72% isodose line covering 95% of the target volume. Since that time, we have reduced hypofractionation doses for meningiomas. Now, we would rather treat this tumor with 5 fractions of 5.5 Gy (mean dose) (equivalent to 5 fractions of 5 Gy of classic CyberKnife prescription to the isodose line, covering 95% of tumor volume).

When treating, we expected several possible complications: severe skin reaction, brain tissue edema, and optic neuropathy. Fortunately, only mild reversible reactions were registered: alopecia (Grade 2 CTCAE 4.02), skin hyperemia (Grade 1, CTCAE 4.02), and oral mucositis (Grade 2 CTCAE 4.02). 

The key features which allowed us to diminish adverse reaction probability are: irradiation conformity and selectivity (which were achieved with multiple non-isocentric, non-coplanar beams and inverse treatment planning); treatment support with medications provided (dexamethasone for possible edema and acetazolamidum for intracranial hypertension); increased interfraction interval; original tumor location, and growth along the brain surface without forming nodes invading the brain. We suggest that increased interfraction interval (for 48 or 72 (for weekend) hours) could also provide additional normal tissue sparing. Meningiomas are characterized by low a/b ratio (around 3.3) [6]. Larger interfraction interval was considered to have little effect on tumor growth control.

The case presented appeared to be unique. However, each year we observe several cases which present similarities to the presented one. These also seem to be treatment challenge. We believe that such cases are not local problems associated with low MRI availability. Rather, the fact of detection of such meningiomas is determined by tumor biology. These subtypes of meningiomas are characterized by slow, long-term asymptomatic tumor growth along the meninges.  

We reviewed published papers and did not find similar cases. Nevertheless, there were successful radiation treatments (radiotherapy and radiosurgery) reported, which still may be considered risky.

Deibert and Kondziolka reported a case of radiosurgical irradiation of a meningioma filling the posterior two-thirds of the superior sagittal sinus [7]. Target volume was not presented. Tumor linear extent composed 16 cm, and transverse dimension composed 15 mm. Six years after Gamma Knife radiosurgical treatment with 12 Gy marginal dose, tumor shrinkage and clinical improvement were obvious. No complications were found.

Ganz, et al. reported a series of 97 patients treated with Gamma Knife radiosurgery for large meningiomas of 10-43 cc (mean - 15.9 cc) with 12 Gy marginal dose [8]. Three cases of radiologic adverse events were registered. Two of them were clinically significant. All reactions were completely reversed after treatment. Despite dose reduction (comparing to commonly used 14 Gy), no tumor growth was detected with a median follow-up period of 54 months (25-86 months).

## Conclusions

We present a case of successful hypofractionated irradiation of a huge hemicranial meningioma with the CyberKnife system. While the outcome in this patient was positive, more experience is essential before we recommend it as an accepted clinical alternative. The suggestion is not to simply copy our experience but to take it into consideration when discussing similar clinical situations.

For decades, the territory of radiosurgery and standard fractionation has been thoroughly investigated. As a result, radiation oncologists and radiosurgeons have stated limitations of practical safe treatment and comfort level when performing it. Overcoming these constraints could increase therapeutic capabilities and allow treating of unusual tumors. For the moment, hypofractionation appears to be “terra incognita”, which still should be discovered and conquered.
